# Prognosis of limited-stage small cell lung cancer with comprehensive treatment including radical resection

**DOI:** 10.1186/s12957-020-1807-1

**Published:** 2020-02-03

**Authors:** Lili Zhong, Jiaojiao Suo, Ya Wang, Jialong Han, Huijie Zhou, Hao Wei, Jiang Zhu

**Affiliations:** 1 Department of Thoracic Oncology, West China Hospital, Sichuan University, Chengdu, China; 2 West China School of Medicine, Sichuan University, Chengdu, China

**Keywords:** Limited-stage small cell lung cancer, Radical surgery, Neoadjuvant chemotherapy, Concurrent chemoradiotherapy, Prognosis

## Abstract

**Background:**

The NCCN (National Comprehensive Cancer Network) Clinical Practice Guidelines in Oncology (NCCN guidelines) recommend radical resection for T1-2N0M0 patients with limited-stage small cell lung cancer (LS-SCLC). However, only about 5% of patients with small cell cancer (SCLC) were initially diagnosed as T1-2N0M0. The purpose of our study was to analyze and compare the effects of the comprehensive treatment including radical surgery and concurrent chemoradiotherapy on the prognosis of patients with LS-SCLC.

**Methods:**

We comprehensively reviewed the medical data of patients with SCLC diagnosed by pathology in our hospital from January 2011 to April 2018. The Ethics Committee of West China Hospital of Sichuan University approved the study. Finally, 50 patients with good follow-up and complete medical data were selected as the surgical group (S group). According to the clinical characteristics of the patients in the S group, 102 LS-SCLC patients who received concurrent chemoradiotherapy in the same period were included in the CCRT group (concurrent chemoradiotherapy group) as the control group. Then according to the orders of the adjuvant treatments, the patients in the S group were divided into the SA group (radical surgery + adjuvant chemotherapy + adjuvant radiotherapy group, 30 cases in total) and the NS group (neoadjuvant chemotherapy + radical surgery + adjuvant chemotherapy ± adjuvant radiotherapy group, 20 cases in total) for subgroup analysis. The SPSS 23.0 software was used for statistical analysis, and the *t* test was used for group comparison; Kaplan-Meier was used for survival analysis. *P* < 0.05 demonstrates a statistically significant difference.

**Results:**

The median progress-free survival (PFS) in the S group (73 months) was significantly better than that in the CCRT group (10.5 months, *P* < 0.0001), and the median overall survival (OS) in the S group (79 months) was also significantly better than that in the CCRT group (23 months, *P* < 0.0001). Subgroup analysis showed that there was no significant difference between the NS group and the SA group.

**Conclusions:**

For LS-SCLC patients, the comprehensive treatment including radical surgery (radical surgery + adjuvant chemotherapy ± adjuvant radiotherapy/neoadjuvant chemotherapy + radical surgery + adjuvant chemotherapy ± adjuvant radiotherapy)may be superior to concurrent chemoradiotherapy.

## Introduction

Lung cancer is the most common cause of cancer and cancer deaths among men worldwide and the second most common cause of cancer death in women worldwide [1, 2]. The pathology of lung cancer is mainly divided into small cell lung cancer (SCLC) and non-small cell lung cancer (NSCLC). SCLC accounts for about 15% of lung cancer patients [2]. Most (about 70%) patients with SCLC are diagnosed as having extensive small cell lung cancer (ES-SCLC). Only about 30% of SCLC patients are diagnosed as having limited-stage small cell lung cancer (LS-SCLC), but their prognosis is still not optimistic with a median survival time of 15–20 months [3]. Since the 1970s, platinum-based combination chemotherapy with etoposide (EP) or irinotecan (IP) has been established as the main treatment of SCLC. For decades, platinum combined with etoposide chemotherapy and combined with concurrent radiotherapy has been the standard treatment for LS-SCLC [4, 5]. Although SCLC is sensitive to radiotherapy and chemotherapy, it is prone to drug resistance. The effect of later line treatment is not optimistic, and the survival time of patients is short [6]. Due to the failure of early clinical studies of surgery in LS-SCLC patients, the NCCN (National Comprehensive Cancer Network) Clinical Practice Guidelines in Oncology (NCCN guidelines) recommend only the patients with LS-SCLC who have staged T1-2N0M0 cancer to undergo radical surgery. However, in the initial diagnosis of SCLC patients, less than 5% meet this criterion [7]. The standard treatment recommended by the current NCCN guidelines for LS-SCLC is concurrent chemoradiotherapy [8], and prophylactic cranial irradiation (PCI) is considered for LS-SCLC patients who have good responses to induction therapy. Although the effective rate of first-line treatment is as high as 80%, most patients would relapse within 6 months after the completion of the initial treatment [9]. Therefore, a better treatment strategy is still our unremitting goal of exploration. This study was intended to analyze and compare the effects of the comprehensive treatment including radical surgery and concurrent chemoradiotherapy on the prognosis of patients with LS-SCLC.

## Methods

### Patients

The Ethics Committee of West China Hospital of Sichuan University approved the study.

From January 2011 to April 2018, patients with SCLC were chosen based on information from the Department of Medical Records West China Hospital of Sichuan University. Then patients with LS-SCLC were selected by consulting medical records. Finally, 152 patients with good follow-ups and complete medical data were included in our study. The primary endpoints were progression-free survival (PFS) and overall survival (OS).

### Inclusion criteria

1. Patients were biopsied by operation/fiber optic/bronchoscope or by endobronchial ultrasound-guided transbronchial needle aspiration (EBUS-TBNA)/mediastinoscopy or pulmonary puncture under image guidance and then diagnosed with SCLC after pathological investigation or pathological consultation at the West China Hospital of Sichuan University.
2. After PET-CT, contrast-enhanced thoracic and abdominal CT, head MRI, bone scan, and other imaging examinations, the patient was diagnosed with LS-SCLC.
3. Patient received anti-tumor treatment for SCLC at the West China Hospital, including surgery and/or chemotherapy and/or radiotherapy.
4. Patient medical records and follow-up data were completed.

### Exclusion criteria

1. Patient received only palliative and symptomatic support treatment and did not receive any treatment or gave up treatment during the course.
2. Patient had severe cardio-cerebrovascular disease or other diseases that may have had a significant impact on prognosis.
3. Patient was lost to follow-up or patient medical records/important information was incomplete or missing.
4. Baseline examination revealed a possible (unidentified) presence of lesions in the patient beyond the limitation period.
5. Patient received targeted therapy.

### Grouping

1. S group (surgical group), 50 patients in total:

- SA group (radical surgery + adjuvant chemotherapy ± adjuvant radiotherapy group), 30 cases in total
- NS group (neoadjuvant chemotherapy + radical surgery + adjuvant chemotherapy ± adjuvant radiotherapy group), 20 cases in total

2. CCRT group (concurrent chemoradiotherapy group), 102 patients in total

### Therapeutic schedule

#### S group (surgical group)

1. SA group (radical surgery + adjuvant chemotherapy ± adjuvant radiotherapy group)
  - Each patient was admitted to the hospital to complete the relevant examination, and the radical operation (lobectomy combined with regional lymph node dissection) was performed after surgical contraindications were excluded. Then adjuvant therapy was arranged according to the results of the postoperative pathological examination.
  - Adjuvant chemotherapy: EP regimen (etoposide + cisplatin, etoposide 100 mg/m^2^ d1–d3 + cisplatin 75 mg/m^2^ d1–d3, repeated every 21 days) or EC regimen (etoposide + carboplatin, etoposide 100 mg/m^2^ d1–d3 + carboplatin AUC=4–6 d1, repeated every 21 days), with a maximum of four cycles.
  - Adjuvant radiotherapy: Patients with lymph node metastasis confirmed by postoperative pathology were treated with adjuvant radiotherapy (mediastinal radiation therapy, 45 Gy, 1.5 Gy bid, 30 fractions for 3 weeks).

2. NS group (neoadjuvant chemotherapy + radical surgery + adjuvant chemotherapy ± adjuvant radiotherapy group)
  - Neoadjuvant chemotherapy (EP or EC regimen same as before) was given to patients after discussion by a multi-disciplinary treatment (MDT) consisting of the Department of Thoracic Surgery and Department of Thoracic Oncology with a maximum of four cycles.
  - Thoracic and abdominal contrast-enhanced CTs were reexamined to evaluate the efficacy. After the MDT discussion, the patients who could be treated surgically were selected for radical surgery (lobectomy with regional lymph node dissection).
  - The Department of Thoracic Oncology then decided if each patient needed adjuvant therapy or not based on postoperative pathology.

#### CCRT group (concurrent chemoradiotherapy group)

Each patient received concurrent chemoradiotherapy (completing radiotherapy within 42 days after the start of chemotherapy). The chemotherapy regimen was EP or EC (same as before). The method was CF (conventional fraction) radiotherapy, with a dose of 60–70 Gy/30–35 times, once a day, 5 days/week.

### Follow-up

West China Hospital’s hospital information system (HIS), telephone, letter, and public security systems were used to collect patient data. Each patient’s basic information was retrieved by the medical records department, and the patient’s medical records and information were reviewed in the HIS system after screening according to the inclusion criteria and the exclusion criteria. Then each patient’s follow-up information was obtained through medical records retrieval, telephone, and inquiry to the public security household registration system.

Follow-up deadline: September 2019.

### Statistical analysis

This study is a single-center retrospective clinical study based on the real world. The SPSS 23.0 software was used for statistical analysis, and the *t* test was used for group comparison; Kaplan-Meier was used for survival analysis. *P* < 0.05 demonstrates a statistically significant difference.

## Results

### Basic information on the 152 patients with LS-SCLC

This study selected patients with SCLC who were diagnosed and treated in West China Hospital of Sichuan University from January 2011 to April 2018. The information was retrieved from the Information Department of West China Hospital of Sichuan University, removing repeated cases (including revisiting and repeated treatment patients). After reviewing each patient’s medical records and imaging data through the HIS, we selected the patients who were diagnosed as LS-SCLC at the time of initial. Each patient’s treatment and survival status were followed up by telephone, medical record system, and public security system. Patients who had unfinished treatment or only accepted supportive treatment or whose medical records were incomplete or missing important information or lost follow-up were excluded. Finally, 152 patients were enrolled in the study. The deadline for follow-up was September 2019.

Thirty patients (19.7%) underwent surgical treatment firstly and then underwent adjuvant therapy or not according to postoperative pathology; 20 patients (13.2%) underwent neoadjuvant chemotherapy firstly and then received surgery. A total of 102 patients (67.1%) did not undergo surgery and received concurrent chemoradiotherapy according to the standard treatment regimen.

Survival analysis was performed on all these 152 patients, and the median PFS was 18 months and the median OS was 30 months. The 1-year survival rate was 78.9% (120/152), the 2-year survival rate was 58.6%(89/152), and the 5-year survival rate was 27.6% (42/152).

**Fig. 1:**
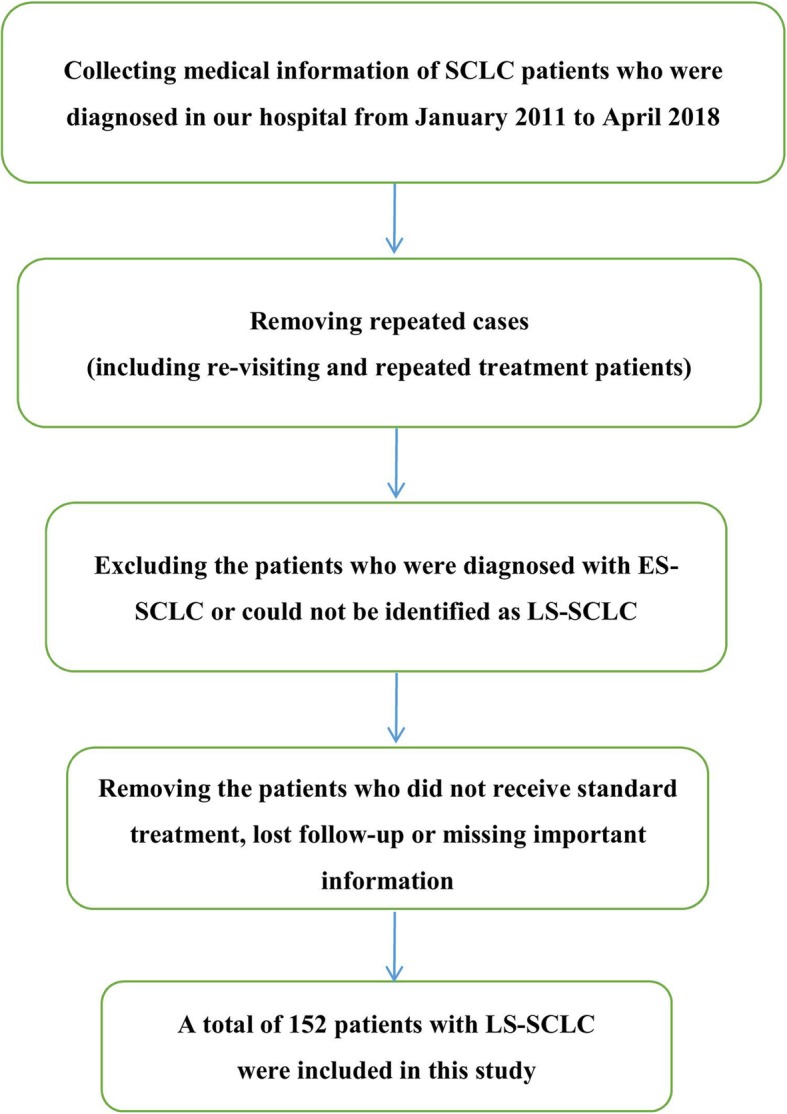
The screening process for patients in this study

The screening process for patients in this study is shown in Fig. 1; basic information on the 152 patients with LS-SCLC is shown in Table 1.

**Fig. 2:**
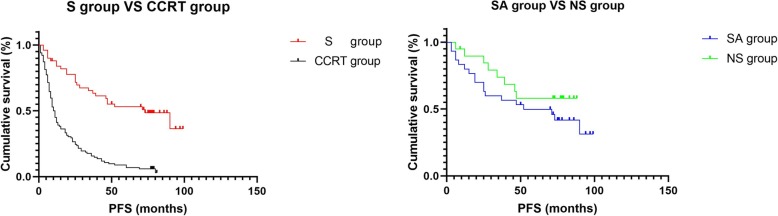
**a** S group vs CCRT group. S group, surgical group; CCRT group, concurrent chemoradiotherapy group. The median PFS of the S group was 73 months, and the median PFS of the CCRT group was 10.5 months. *P* < 0.0001 demonstrates a statistical difference. **b** SA group vs NS group. SA group, radical surgery + adjuvant chemotherapy ± adjuvant radiotherapy group; NS group, neoadjuvant chemotherapy + radical surgery + adjuvant chemotherapy ± adjuvant radiotherapy group. The median PFS of the SA group was 52 months, and the median PFS of the NS group was not reached. *P* = 0.252 demonstrates no statistical difference

**Table 1:** Basic information on the 152 patients with LS-SCLC

Item	Classification	S group	CCRT group	*P*
Total number of cases		50	102	
Age (years old)	≥ 60 < 60	14 36	45 57	0.097
Gender	Male Female	41 9	74 28	0.232
Primary tumor site	Left lung Right lung	24 26	54 48	0.607
Basic diseases (hypertension/diabetes)	No Yes	40 10	77 25	0.550
TNM staging	I II III	25 9 16	50 19 33	0.937
Smoking history	No Yes	31 19	48 54	0.088
PCI	No Yes	12 38	35 67	0.262
Chemotherapy cycles	≥ 4 < 4	37 13	91 11	0.069

*S group* surgical group, *CCRT group*, concurrent chemoradiotherapy group

### Survival

#### Progression-free survival (PFS)

The relationship between the PFS curve and the treatment modality is shown in Fig. 2a, b.

#### Overall survival (OS)

**Fig. 3:**
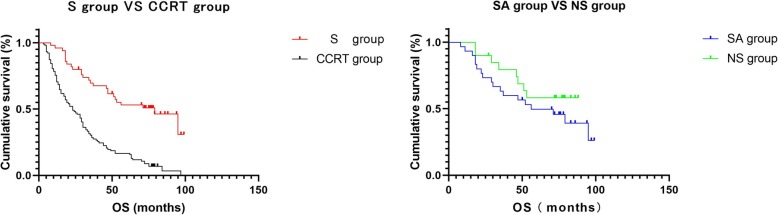
**a** S group VS CCRT group. S group, surgical group; CCRT group, concurrent chemoradiotherapy group. The median OS of the S group was 79 months, and the median OS of the CCRT group was 23 months. *P* < 0.0001 demonstrates a statistical difference. In the S group, the 1-year survival rate was 96.0%, the 2-year survival rate was 80.0%, and the 5-year survival rate was 28.0%. In the CCRT group, the 1-year survival rate was 70.6%, the 2-year survival rate was 48.0%, and the 5-year survival rate was 16.7%. **b** SA group VS NS group. SA group, radical surgery + adjuvant chemotherapy ± adjuvant radiotherapy group; NS group, neoadjuvant chemotherapy + radical surgery + adjuvant chemotherapy ± adjuvant radiotherapy group. The median OS of the SA group was 56 months, and the median OS of the NS group was not reached. *P* = 0.266 demonstrates no statistical difference

The relationship between the OS curve and the treatment modality is shown in Fig. 3a, b.

## Discussion

Lung cancer was very rare before the twentieth century, but due to environmental changes and genetic susceptibility the incidence and mortality has jumped to top among all cancers in the world [10, 11]. In 2012, there were 1.8 million new lung cancer cases and 1.6 million lung cancer deaths worldwide accounting for 19% of all cancer deaths [12]. Lung cancer is divided into SCLC and NSCLC. SCLC develops rapidly and has strong invasiveness and poor prognosis. About 30% of SCLC patients are diagnosed with LS-SCLC at the time of diagnosis and the median OS is 15–20 months. The remaining 70% SCLC patients are diagnosed as ES-SCLC at the time of diagnosis and the median OS is only 8–13 months [12–14]. Because most LS-SCLC patients have regional lymph node metastasis at the time of diagnosis, radical surgery has been considered a surgical contraindication while chemotherapy is the main treatment. There are some studies that have overturned the status of surgery in patients with this disease [15, 16]; however, these studies did not screen for TNM staging in the LS-SCLC patients and the patients only underwent radical surgery without any adjuvant. But some previous studies showed that some patients with early-stage SCLC may benefit from surgery [17, 18]. At present, the NCCN guidelines recommend only the patients with LS-SCLC who have staged T1-2N0M0 to undergo radical surgery and concurrent chemoradiotherapy is the standard treatment model for other LS-SCLC patients [19]. Although SCLC is sensitive to chemotherapy, it is prone to drug resistance which leads to treatment failure and poor disease outcome [20]. In this article, we analyzed and compared the effects of the comprehensive treatment including radical surgery and concurrent chemoradiotherapy on the prognosis of patients with LS-SCLC. The results indicated that the surgery plays an important role in the treatment of patients with LS-SCLC and that comprehensive treatment involving surgery can significantly improve the prognosis of patients compared with concurrent chemoradiotherapy.

Compared with adjuvant chemotherapy combined with surgery, some previous studies have shown that neoadjuvant chemotherapy combined with surgery may could give the LS-SCLC patients a better chance of survival [21–23]. This result may be attributed to the effect of Stage T or N down-staging by neoadjuvant chemotherapy, which helps patients diminish several symptoms and obtain more survival benefit from radical resection [21]. But the controversy about neoadjuvant chemotherapy for LS-SCLC still exists. There also have studies which showed that compared with adjuvant chemotherapy combined with surgery neoadjuvant chemotherapy combined with surgery has no better survival [24]. Though our study demonstrates that there is no statistically significant difference between the two subgroups a trend shows that neoadjuvant chemotherapy combined with surgery may have the better chance of survival than adjuvant chemotherapy combined with surgery.

Because there were a relatively few number of patients in this study and it is a retrospective study, the general situations of the patients involved were not that balanced. There are also other inevitable defects such as the mixed bias caused by the heterogeneity of the social and economic conditions of the group, as well as evitable selection bias. All of that may have affected the effectiveness of this study. The efficacy of the comprehensive treatment including radical surgical for LS-SCLC patients needs to be confirmed with a larger sample size and multi-center research results.

## Conclusions

For LS-SCLC patients, the comprehensive treatment including radical surgery (radical surgery + adjuvant chemotherapy ± adjuvant radiotherapy/neoadjuvant chemotherapy + radical surgery + adjuvant chemotherapy ± adjuvant radiotherapy) may be superior to concurrent chemoradiotherapy.

## Data Availability

All data came from the Department of Medical Records, West China Hospital of Sichuan University.
